# Novel force feedback technology improves suturing in robotic-assisted surgery: a pre-clinical study

**DOI:** 10.1007/s00464-024-11472-9

**Published:** 2024-12-30

**Authors:** Elliot L. Servais, Laila Rashidi, Priyanshi Porwal, Mark Garibaldi, Andrew J. Hung

**Affiliations:** 1 Lahey Hospital and Medical Center, UMass Chan Medical School, Burlington, MA USA; 2 MultiCare Health System, Seattle, WA USA; 3 Intuitive Surgical, Sunnyvale, CA USA; 4 Cedars-Sinai Medical Center, Los Angeles, CA USA

**Keywords:** Surgical training, Force feedback, Suturing

## Abstract

**Introduction:**

The inability to sense force applied to tissue is suggested as a limitation to robotic-assisted surgery (RAS). This pre-clinical study evaluated the impact of a novel force feedback (FFB) technology, integrated on a next-generation robotic system that allows surgeons to sense forces exerted at the instrument tips, on suturing performance by novice surgeons during RAS.

**Methods:**

Twenty-nine novice surgeons (< 50 RAS cases in the last 5 years) were randomized into two groups with (*n* = 15) or without (*n* = 14) FFB sensing. Participants performed interrupted stitches on ex vivo porcine bladder and running stitches on porcine aorta (Fig. 1A) over four runs. Average forces applied, number of errors, time for exercise completion, and Robotic Anastomosis Competence Evaluation (RACE) technical skill ratings were compared using a three-way mixed-model ANOVA and applicable post hoc tests.

**Fig. 1:**
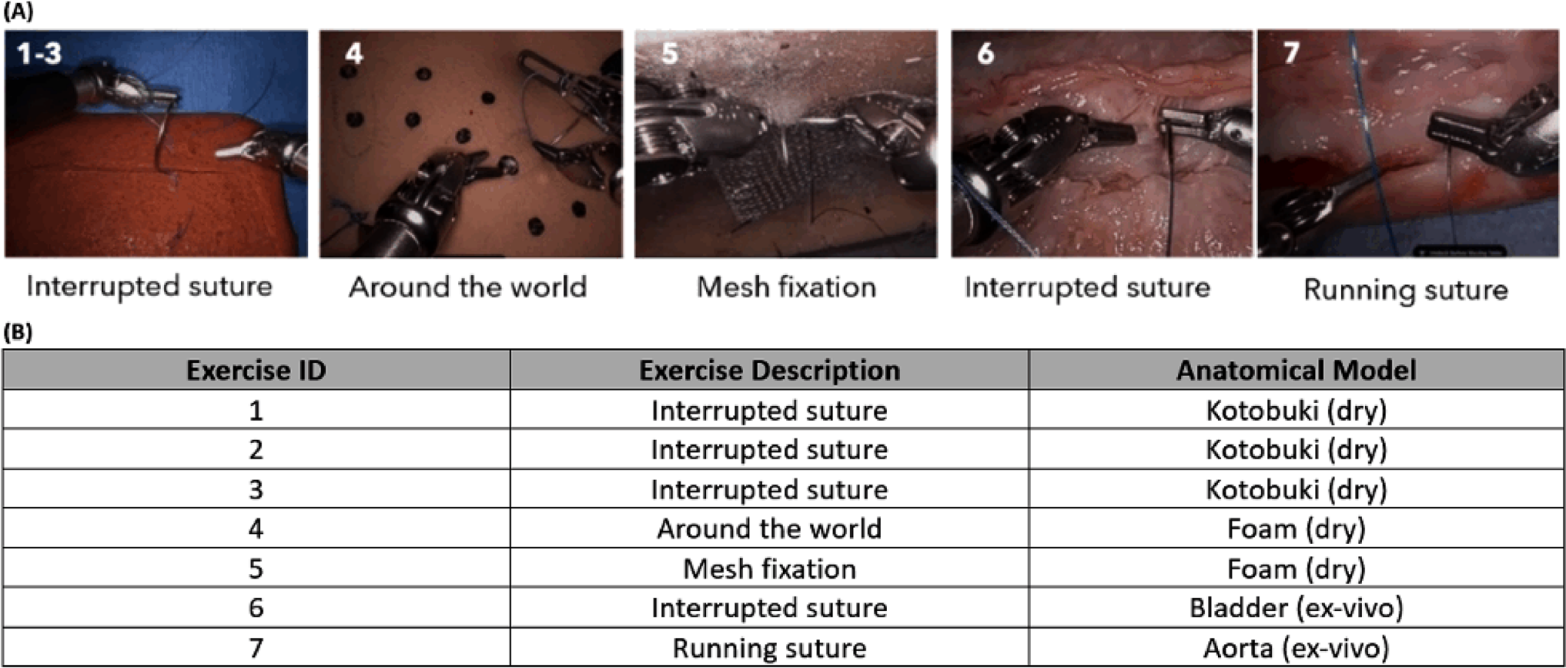
**A** View from surgeon console of participant performing suturing tasks using Kotobuki dry model (Exercise 1 to 3), Foam dry model (Exercise 4 and 5), Urinary Bladder ex vivo tissue (Exercise 6), and Aorta ex vivo tissue (Exercise 7). **B** Description of 7 suturing exercises and anatomical models used for each exercise

**Results:**

FFB sensing significantly lowered the mean force applied (bladder, 1.71 N vs 2.40 N, *p* < 0.006; aorta, 1.80 N vs 2.53 N, *p* < 0.006), average number of errors (bladder, 0.59 vs 1.76, *p* < 0.001; aorta, 0.38 vs 1.14, *p* < 0.001), and the time to completion (bladder, 659 s vs 781 s, *p* = 0.002; aorta, 460 s vs 570 s, *p* = 0.001) (Fig. 1C). The FFB group applied less tissue trauma with a higher RACE skill score (3.75 vs 3.03, *p* = 0.012).

**Conclusion:**

This study showed that novice surgeons using FFB-enabled instruments completed suturing tasks using less force, with fewer errors, taking less time, and less tissue trauma during RAS. Future studies are required to better understand the impact of FFB technology on surgical performance and potential patient benefits.

Robotic-assisted surgery (RAS) is a minimally invasive surgical approach with well-recognized technological and operative benefits [1–8]. However, a perceived limitation of minimally invasive surgery such as traditional laparoscopic surgery or RAS is the absence of haptic feedback. “Haptics” describes touch feedback in general, which may include both kinesthetic feedback (force feedback) [9] and cutaneous feedback (tactile feedback) [10, 11]. “Force feedback” refers to the kinesthetic feedback provided to the user’s hands at the hand controllers based on the forces measured at the tip of the instrument [6, 12, 13]. Absence of haptic feedback may lead to unintentional application of strong forces on tissue that could increase the risk of tissue injury, and inadequate suturing, such as air knots or suture breakage. These risks could ultimately translate to prolonged operative time and increased patient morbidity [2, 4, 12–16].

Currently, surgeons performing minimally invasive surgery counterbalance for the lack of haptic feedback by developing necessary motor skills [16] while relying on visual haptics to estimate the forces applied to tissues [5]. Acquired through practice, visual haptics is the ability to estimate forces applied on tissues based only on visually apparent cues [13]. Haptic input to visual feedback may improve tissue characterization, particularly in relation to tension placed on tissue during surgery. The augmentation of conventional visual information, with haptic feedback has shown improved surgical performance in laparoscopic and robotic surgery [17–19].

Surgical tasks such as suturing are particularly difficult to perform without the haptic sense because excessive forces can break delicate sutures or injure healthy tissue, while insufficient forces might cause slippage or loose sutures [6, 13, 14, 20]. Force feedback is, therefore, a crucial component that could enhance the performance of surgeons and their suturing proficiency [7, 21, 22]. Studies have shown that the combination of visual haptics and force feedback can potentially reduce the force applied on tissues during suturing, thereby improving the surgeon’s control of exerted force, and conceivably leading to improved suturing performance in robotic surgery [1, 13, 16, 18, 21, 23]. Therefore, being able to sense the forces applied to tissue using force feedback instruments is hypothesized to be useful for surgeons to perform common surgical tasks.

This pre-clinical study evaluates the use of a force feedback technology that has been developed and integrated into the new generation of the da Vinci robotic surgical system, “da Vinci 5” (Intuitive Surgical, Sunnyvale, CA). This technology involves the use of instruments with built-in force sensors that allow the surgeons to sense at their hand controllers the forces applied at the instrument tips. This study tests the hypothesis that the use of force feedback improves the force applied, error rate, time to completion, quality of technique, and mental demand of suturing tasks during robotic-assisted surgery among novice surgeons in the early phase of assimilating to robotic surgery (Figs. 2, 3).

**Fig. 2:**
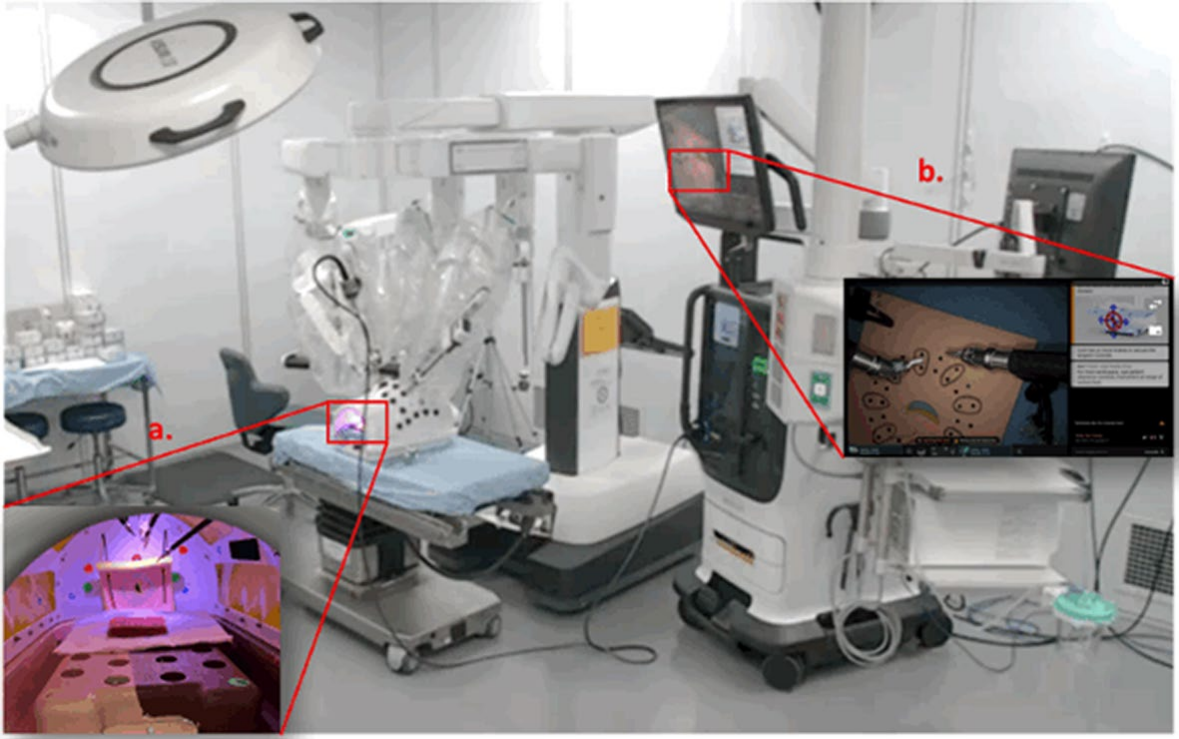
Robotic System Setup. Inset **a**: Kotobuki model placed inside the abdominal body model. Inset **b**: Endoscopic view of the setup with two force feedback Large Needle Drivers inserted

**Fig. 3:**
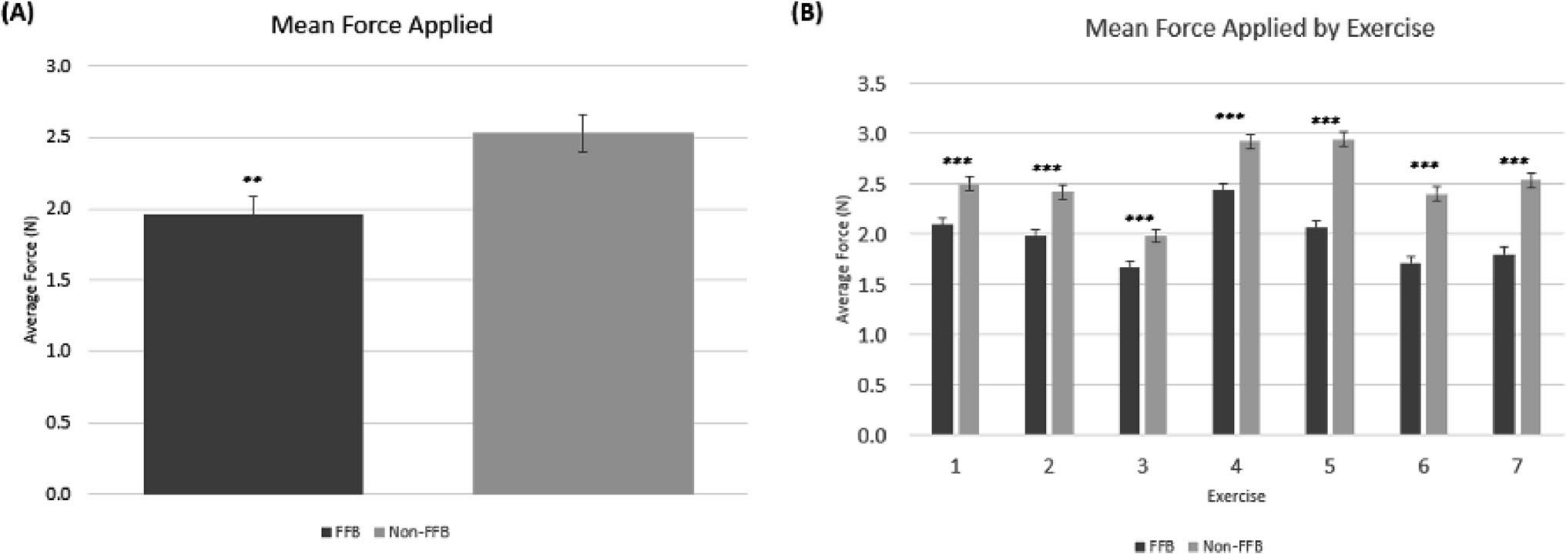
**A** Overall mean force applied on tissues by the surgeons in the FFB and non-FFB cohort **B** Mean force applied on tissues by surgeons in the FFB and non-FFB cohort across each exercise. * = *p* < 0.05; ** = *p* < 0.01; *** = *p* < 0.001

## Methods

### Study participants

Twenty-nine novice surgeons who had completed < 50 RAS procedures in the last five years were recruited to participate in this study. Of the 29 participants, 26 were general surgeons, two surgeons specialized in urologic/gynecologic surgery, and one surgeon specialized in thoracic surgery. Surgeons who had prior experience with da Vinci 5 Surgical System and prior use of force feedback instruments were excluded from the study.

### Study design

In this single-blinded, between-subjects study, surgeons were randomly assigned to one of the two study cohorts: those who performed suturing exercises using force feedback (FFB; *n* = 15) or those who performed suturing without force feedback (non-FFB; *n* = 14). The surgeons used instruments with built-in force sensors. These newly developed FFB instruments were similar in form and function to the standard instruments used in RAS, except for the addition of force sensors at the instrument tips that measured push or pull forces (along the X, Y, and Z axes) and transmitted these forces to the hand controllers at the surgeon console allowing the integration of force sensing with the use of the surgical system. The FFB instruments do not provide grip feedback or tactile sensation. The feedback relayed to the surgeon’s hand controls can be scaled to three selectable sensitivity settings, namely ‘Low,’ ‘Medium,’ or ‘High.’ For this study, ‘Medium’ was set as the default sensitivity setting. The non-FFB cohort performed the suturing tasks using the same instruments with the sensors, with the setting at the hand controllers set to the ‘Off’ position.

### Models

This study used both dry models and ex vivo porcine tissue models (Fig. 1). The dry models consisted of either a plant-based simulated organ called VTT RBC Type (Kotobuki Medical, Japan) or a foam suture pad. The ex vivo tissue models included porcine aorta and urinary bladder (Yosemite Foods Inc., Stockton, CA).

### Suturing exercises

Each surgeon performed seven specialty-agnostic suturing exercises (Ex) repeated four times (over Runs 1 through 4), with a total of 28 suturing sessions per surgeon. The dry model exercises included interrupted sutures, mesh fixation, and needle driving (Ex 1 through 5). In exercises 1 through 3, surgeons tied a knot across the incision line of the Kotobuki model using three different sutures. Exercise 4 involved driving the needle out-to-in, clockwise, through eight pairs of designated targets on a foam pad. This was followed by Exercise 5 that involved approximating the mesh to the foam model using a running suture technique. Exercises 6 and 7 were performed on ex vivo porcine tissues. Exercise 6 involved reapproximating an incision in the bladder with a minimum of six interrupted sutures and exercise 7 involved the repair of an aortic defect using a running stitch (Fig. 1).

### System setup and test instruments

For the study, da Vinci 5 Surgical System (Intuitive Surgical Inc., Sunnyvale, California, USA) and its accessories—such as drapes, cannulas, and seals—were employed. For dry exercises, the system was docked to an abdominal body model with the Kotobuki model or foam suture pad fixed to the base (Exercise 1, 2, 3, and 4) or the ceiling of the model (Exercise 5) depending on the exercises. For the ex vivo tissue exercises, the defects on the tissue were created at the beginning of the day and placed on trays. The system was positioned above the tissue tray at standardized working distance and air docked. Two 8-mm Force Feedback Large Needle Drivers and a 30-degree Endoscope were inserted through the cannulas and were used by participants for performing the suturing tasks. For both FFB and non-FFB participants, Force Feedback was set to Off for Run 1 to collect baseline data. For FFB participants, the default Force Feedback setting was set to Medium for all exercises during runs 2, 3, and 4. For non-FFB participants, Force Feedback was set to Off for all exercises during runs 2, 3, and 4. The motion scaling was set to Fine (7:1) for all participants. The surgeons could choose their ergonomic settings on the surgeon console to suit their comfort level including the position of headrest, foot pedal tray, height of endoscopic viewer etc. The endoscope settings like brightness and contrast were also not controlled as a part of the study. Handheld 5-mm laparoscopic grasper and scissors were used for mid-exercise suture exchange and cutting tasks. The above-mentioned system setup is shown in (Fig. 2)

### Experimental procedure

Each surgeon completed four runs comprised seven simulated exercises as described in the following standardized steps: (1) surgeons received an overview of the study prior to its commencement; (2) surgeons in the FFB cohort received a product training on Force Feedback instruments and orientation to the new robotic system, while surgeons in the non-FFB cohort received a refresh product training on standard instruments and an orientation to the new robotic system. At the conclusion of training, all surgeons practiced two interrupted suture tasks. The first practice attempt included coaching from the study moderator and the second attempt did not include coaching from the moderator; and (3) surgeons completed four runs within their respective condition.

### Performance metrics and data analysis

Throughout each run, the quality of clinical technique, head-in following time, and mental load were measured to assess differences in surgeon performance:

### (a) Metrics

1. **Clinical technique**
  i. ***Mean force applied***: The forces applied on tissues by the Force Feedback instruments during different tasks were measured. The magnitudes of forces were directly collected and stored in the system data log of the robotic system. A MATLAB script was used to summarize the force data collected from the system data logs between exercise start and stop points. Data included average, max, min force measures, timestamp, console time duration at a particular setting, etc. The measured forces were averaged across the two Needle Drivers. Forces less than 0.5 N were excluded as 0.5 N is the typical minimum force threshold at which users of the robotic system can detect forces at the system hand controllers
  ii. ***Error count***: The number of errors for each of the seven pre-identified types of errors were recorded across each exercise with time stamps. The following types of errors were counted: needle misdrives, suture fray, suture breaks, needle bend, air knot, inaccurate tension, and tissue/tail pull through.
  iii. ***Technical skills assessment***: Robotic Anastomosis Competency Evaluation (RACE) urethrovesical anastomosis (UVA) that objectively measures surgical performance and skills [24–26]. The evaluation covers six areas of technical suturing proficiency: needle position, needle entry, needle driving and tissue trauma, suture placement, tissue approximation, and knot tying. Measurement of suture quality was based on two suturing tasks involving ex vivo tissue (exercise 6 and 7).
2. **Operational time:** The total amount of time a surgeon’s head was positioned in the surgeon console while actively controlling the instruments to finish the task. Measurement of time was recorded across each exercise.
3. **Mental load/task load index:** Surgeon’s mental load across six dimensions (mental demands, physical demands, temporal demands, task complexity, situational stress, and distractions) was assessed after runs 1 and 3 were completed based on the Surgical Task Load Index (SURG-TLX) [27].

### (b) Data recording

Force data from force feedback instruments was recorded for both FFB and non-FFB participants for all exercises using a MATLAB Graphic User Interface (GUI) by the lab coordinator. This GUI also had built-in stopwatch that was used to record the task completion time for each exercise. The error counts for each exercise were recorded in real time on an Excel worksheet tool by the lab coordinator. The endoscope view was also recorded for future assessment to aid in technical skills analysis.

### (c) Statistical analysis

All statistical analyses were completed using NCSS statistical software. Omnibus three-way mixed-model analysis of variance (ANOVA) was performed (condition x exercise x run) for each metric. Additional omnibus two-way mixed-model ANOVAs (condition x exercise), applicable post hoc tests or independent-samples t tests (for FFB v/s non-FFB condition), and paired sample t test (for time on task) was performed for processing data of baseline (Run 1) and Runs 2–4. One sided p values were calculated for all comparisons. For overall comparisons, a p value < 0.05 was considered statistically significant. For comparisons across exercises and error types, Bonferroni corrections were applied to the p values (e.g., 0.05/7 = 0.007 for a family of 7 comparisons) to account for Type 1 errors. Therefore, a p < 0.007 was considered statistically significant for these comparisons.

### Examination of baseline differences

Before fully analyzing the data, applicable measures collected during Run 1 (baseline, with FFB off) were compared between the two cohorts (FFB and non-FFB) to examine whether pre-existing differences existed across experimental and control groups. No significant baseline differences were observed that required controls or covariates in subsequent analyses.

### Inter-rater reliability of technical skill ratings

Three independent raters reviewed and rated the quality of clinical technique for Exercises 6 and 7 and their ratings were found to be reliable (Cronbach’s *α* > 0.7) based on Intraclass Correlation Coefficients (ICCs) [28] across dimensions and exercises. Thus, RACE ratings were averaged across raters and overall scores were computed by averaging across dimensions.

## Results

### Mean force applied

The overall mean force for all the exercises combined applied by the surgeons was significantly reduced when given force feedback capability (1.96 N with FFB vs 2.53 N, non-FFB; *p* = 0.005; Fig. 3A). Furthermore, the mean force applied by surgeons in the FFB cohort was significantly lower than that of surgeons in the non-FFB cohort across each individual exercise (*p* < 0.001; Fig. 3B).

### Error count

**Fig. 4:**
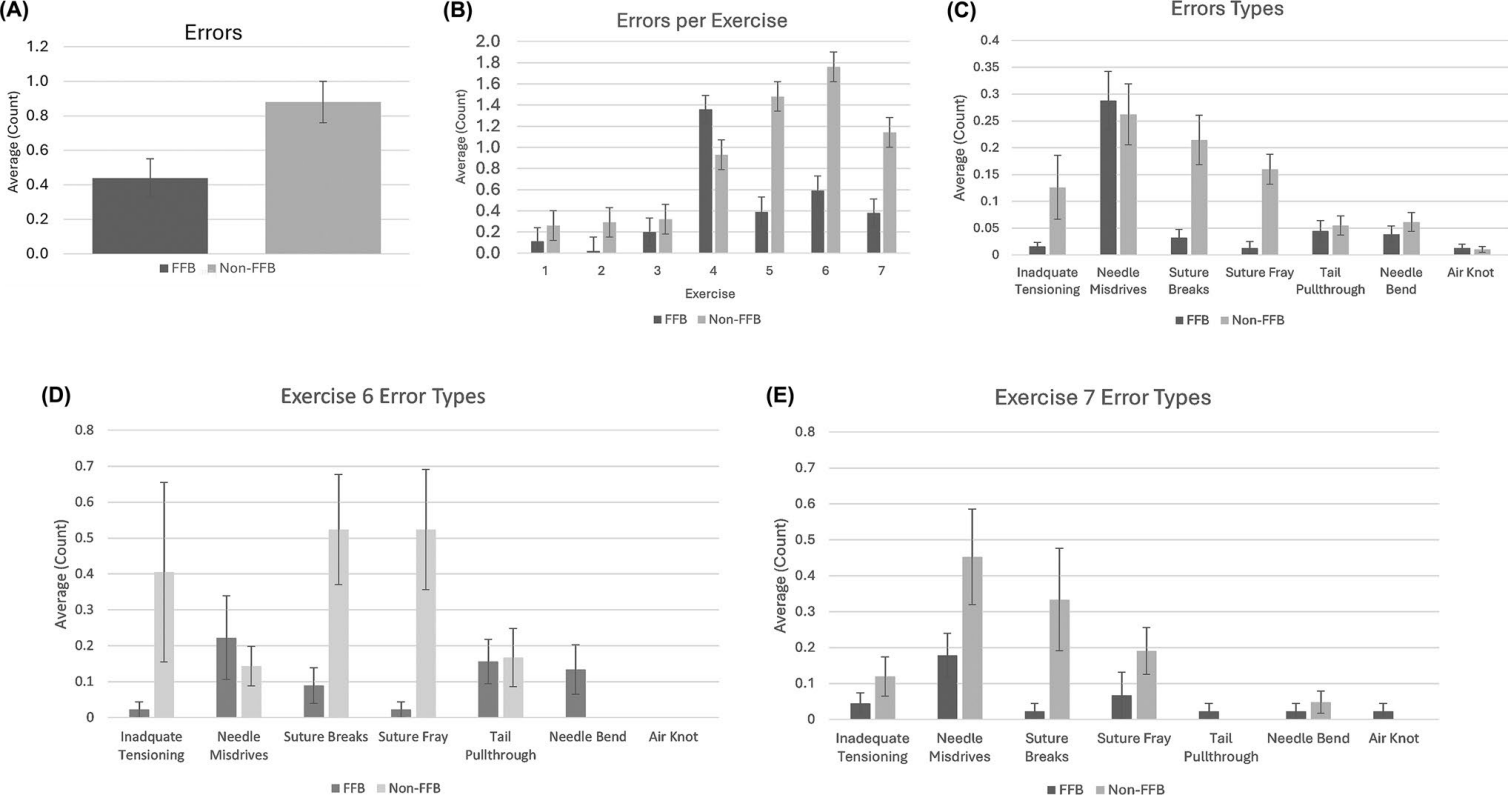
**A** Average error count for all suturing tasks combined. **B** Average error counts observed while performing suturing tasks across each exercise. **C** Average error counts for each of the different error types observed while performing suturing tasks. **D** Average error counts for each of the different error types for only Exercise 6 (ex vivo bladder model). **E** Average error counts for each of the different error types for only Exercise 7 (ex vivo aorta model). * = *p* < 0.05; ** = *p* < 0.01; *** = *p* < 0.001

The average number of errors for all the exercises combined was significantly reduced when force feedback was utilized (0.44 vs 0.88 errors; *p* = 0.012; Fig. 4A). Out of the seven error types that were recorded, the counts of suture break (*p* = 0.001) and suture fray (*p* = 0.0001) were significantly lower in the FFB group as compared to the non-FFB group Fig. 4C). While all exercises showed numerically lower error counts for the FFB cohort compared to the non-FFB cohort, the differences were statistically significant only for exercises 2 and 5 (*p* < 0.001; Fig. 4B) as well as the ex vivo tissue exercises 6 and 7 that were conducted on porcine aorta and bladder (*p* < 0.001; Fig. 4B). Average count of errors for each error type were also analyzed separately for Exercises 6 (Fig. 4D***)*** and 7 (Fig. 4E***)***. Significant differences were found between the FFB and non-FFB cohort for suture fray in Exercise 6 (*p* < 0.006; Fig. 4D).

### Operational time

**Fig. 5:**
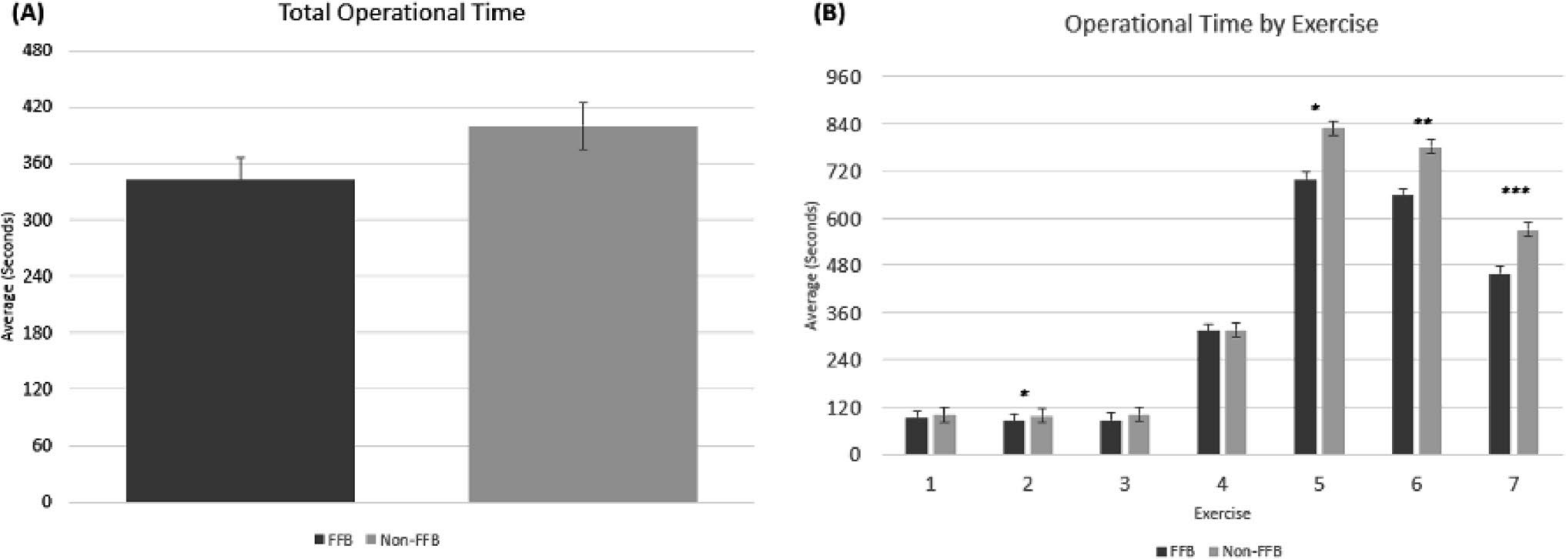
**A** Overall operational time to complete suturing tasks for surgeons in the FFB and the non-FFB cohort. **B** Total time taken to complete suturing tasks across each exercise. * = *p* < 0.05; ** = *p* < 0.01; *** = *p* < 0.001

For all the exercises combined, while the surgeons in the FFB cohort showed a decrease in the overall time to complete the suturing tasks compared to surgeons in the non-FFB cohort, the difference was not statistically significant (Fig. 5A); however, when the data were segregated for each exercise, the time to completion was significantly shorter for the FFB group in dry model exercises 2 and 5, as well as the ex vivo exercises 6 and 7 as shown in Fig. 5B (*p* = 0.015 for Ex 2 and 5; *p* = 0.002 for Ex 6; *p* = 0.001 for Ex 7).

### Technical skills scores

**Fig. 6:**
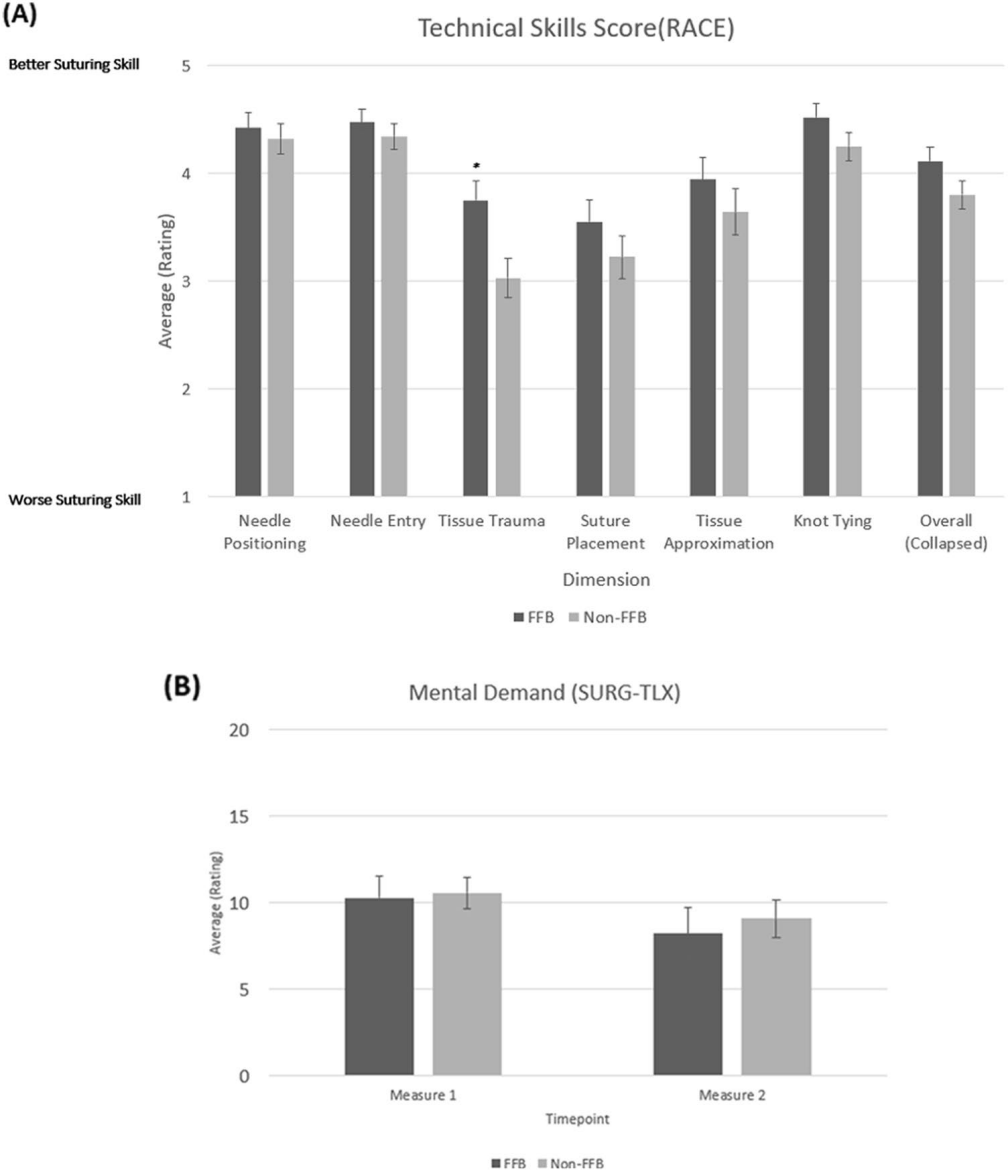
**A** Suturing skill (RACE) ratings across all dimensions of assessment for ex vivo exercises 6 and 7 involving Bladder and Aorta **B** Mental demand ratings as assessed by the SURG-TLX assessment tool measured at two timepoints during the study: Measure 1 and Measure 2. Measure 1 was taken after Run 1, while Measure 2 was taken after Run 3

Figure 6A shows the comparison between the FFB and non-FFB cohorts of the overall RACE scores and the RACE scores for each of the six suturing skills assessed. The results show that the RACE rating for the dimension assessing *tissue trauma* was significantly higher in the FFB cohort compared to the non-FFB cohort (3.75 with FFB vs 3.03 non-FFB; *p* = 0.012), signifying less tissue trauma with the use of FFB.

### Surgeon cognitive task load

The SURG-TLX ratings were collected from surgeons at two timepoints during the study: Measure 1 and Measure 2. Measure 1 was taken after the first Run (baseline – FFB off for both groups), and Measure 2 was taken after the third Run. At Measure 2, surgeons utilizing FFB reported a significant reduction in Mental Demand when compared to Measure 1 (*p* = 0.006; Fig. 6B). However, there were no significant differences between the FFB and non-FFB cohorts in SURG-TLX ratings at both time points measured.

## Discussion

In RAS surgery, application of excessive force can lead to intraoperative organ injury due to pressing or tearing of tissue [16, 29]. In the absence of technology to enable force feedback, the status quo in robotic-assisted surgery is using visual cues acquired through training and experience to estimate applied forces [5, 30–36]. For novice RAS surgeons, this poses a challenge with acclimating to robotic surgery especially for approximating the tension of suture for complex tasks like knot tying. The quality of suture ligation in RAS has been widely evaluated [30, 37–39], but there is a need for more comprehensive studies to thoroughly understand the effects force feedback on the quality of suturing and to determine the optimal amount of force required during RAS. In this study, we found that novice surgeons completed suturing tasks while applying less force on tissue when using force feedback technology. Moreover, the use of force feedback significantly reduced suturing errors and therefore, also reduced tissue trauma in ex vivo tissue exercises. We also saw a significant improvement in operational time with the use of force feedback.

This is the first study to present findings on the impact of force feedback fully integrated with a robotic surgical system on quality of clinical technique, time on task, force applied, and mental load during complex suturing tasks. In prior work, Yamasaki et al. [40] demonstrated the influence of force feedback function on suturing with an experimental robotic prototype involving surgeons who varied in clinical experience. Results showed a 0.6-N reduction in maximum force applied to tissue with force feedback (*p* < 0.001) which was comparable to results from this investigation that revealed a 0.57-N difference in overall mean force applied to tissue between the FFB and non-FFB cohort (*p* < 0.001). This alignment corroborates the expected impact of force feedback on surgeon performance; even though Yamasaki et al. study did not include (a) clinically representative anatomical models in the simulated test cases, (b) experimental control over confounding variables, and (c) robotic surgical system that features a higher threshold for detecting applied forces compared to da Vinci 5.

Past studies have used various experimental models to assess the quality of suturing, yet a standardized model does not exist. This study measured the quality of suturing on ex vivo tissue using RACE, a qualitative assessment tool widely referenced in literature that involves the assessment of clinically relevant qualitative parameters associated with suturing e.g., tissue approximation, knot tying. We observed significant improvements with the FFB cohort, especially with ex vivo tissue exercises. In addition to the reduction in total error count (across all exercises) for the FFB cohort, there was a significant reduction in suture breaks and suture fray (*p* < 0.001) which is associated with the amount of tension applied to suture [14]. Also, we observed a significant reduction in tissue trauma (*p* = 0.012) which is qualitatively associated with the amount of tension applied to suture or retraction when grasping tissue [29]. These results indicate that the use of force feedback technology has the potential to facilitate the completion of complex suturing tasks with improved clinical outcomes.

An important consideration from a surgical standpoint, is how an increase in surgical performance affects operational time. The trade-off between accuracy and efficiency in motor tasks is well studied in psychological sciences [41] but has been only broadly investigated in the context of robotic-assisted surgery [42, 43]. This study showed that surgeons with force feedback sensing had significantly shorter operational times for both ex vivo suturing tasks. This is consistent with investigations that demonstrated how force feedback can improve both accuracy and task completion time by replacing the need for sensory substitution that may require additional processing time by the surgeon [44, 45].

Force feedback may facilitate reductions in task load among surgeons new to robotic-assisted surgery. Surgeons using force feedback technology reported a significant reduction in mental demand after Run 3 relative to Run 1 (baseline); relative to surgeons with non-FFB instruments reported no reductions between these two measures. Future studies that continuously measure surgeon cognitive load while performing surgical tasks may provide more insight into differences in task load over time.

### Limitations

While the study findings suggest that force feedback can improve suturing performance in novice surgeons, there are limitations of the study. First, the errors were counted in real time using a data spreadsheet by clinical subject matter experts. Although no inter-rater reliability was established for errors, all seven types of errors were clearly defined in the study protocol and all coordinators had appropriate clinical training associated with the clinical protocol. The baseline data captured during Run 1 showed that surgeons who were randomly assigned to the FFB cohort applied marginally lower mean force (Mean = 2.28, Standard Deviation = 0.16) compared to surgeons who were randomly assigned to Non-FFB cohort (Mean = 2.73, Standard Deviation = 0.17). No significant main effects or interactions involving baseline were found for any other metric (all other p’s > 0.155).

### Future scope

This study design reflected a narrow focus on surgeon performance with force feedback instruments. Future research should focus on how force feedback influences patient outcomes through surgeon performance and why force feedback enhances skill acquisition related to surgeon proficiency in robotic-assisted surgery. For instance, potential investigations focused on skill acquisition and motor learning [45] could consider whether there are mediating variables augmented by force feedback.
