# Correction: Novel force feedback technology improves suturing in robotic-assisted surgery: a pre-clinical study

**DOI:** 10.1007/s00464-025-11549-z

**Published:** 2025-01-21

**Authors:** Elliot L. Servais, Laila Rashidi, Priyanshi Porwal, Mark Garibaldi, Andrew J. Hung

**Affiliations:** 1 Lahey Hospital and Medical Center, UMass Chan Medical School, Burlington, MA USA; 2 MultiCare Health System, Seattle, WA USA; 3 Intuitive Surgical, Sunnyvale, CA USA; 4 Cedars-Sinai Medical Center, Los Angeles, CA USA

**Correction to: Surgical Endoscopy** 10.1007/s00464-024-11472-9

The original online version of this article was revised to correct error bars in the artwork for Fig. 4, and to add information to the Acknowledgments section.

The original article has been corrected.

