# Correction: Novel force feedback technology improves suturing in robotic-assisted surgery: a pre-clinical study

**DOI:** 10.1007/s00464-025-11647-y

**Published:** 2025-03-05

**Authors:** Elliot L. Servais, Laila Rashidi, Priyanshi Porwal, Mark Garibaldi, Andrew J. Hung

**Affiliations:** 1 Lahey Hospital and Medical Center, UMass Chan Medical School, Burlington, MA USA; 2 MultiCare Health System, Seattle, WA USA; 3 Intuitive Surgical, Sunnyvale, CA USA; 4 Cedars-Sinai Medical Center, Los Angeles, CA USA

**Surgical Endoscopy (2024) 39:1217–1226** 10.1007/s00464-024-11472-9

The original online version of this article was revised to add the following acknowledgement:

We would like to express our sincere appreciation to Komal Singh and Yan Zhong for their assistance with drafting the manuscript, and to Andrew Penfold and Katie Ewing for their contributions to the study design and publication efforts.

The original article has been corrected.

